# Specification and Patterning of *Drosophila* Appendages

**DOI:** 10.3390/jdb6030017

**Published:** 2018-07-14

**Authors:** Mireya Ruiz-Losada, David Blom-Dahl, Sergio Córdoba, Carlos Estella

**Affiliations:** Departamento de Biología Molecular and Centro de Biología Molecular Severo Ochoa, Universidad Autónoma de Madrid (UAM/CSIC), Nicolás Cabrera 1, 28049 Madrid, Spain; mireya.ruiz@cbm.csic.es (M.R.-L.); david.blom-dahl@cbm.csic.es (D.B.-D.); scordoba@cbm.csic.es (S.C.)

**Keywords:** *Drosophila*, appendages, imaginal discs, transcription factors, signaling pathways, proximodistal axis, wing, leg, antenna, appendage primordia

## Abstract

Appendages are external projections of the body that serve the animal for locomotion, feeding, or environment exploration. The appendages of the fruit fly *Drosophila melanogaster* are derived from the imaginal discs, epithelial sac-like structures specified in the embryo that grow and pattern during larva development. In the last decades, genetic and developmental studies in the fruit fly have provided extensive knowledge regarding the mechanisms that direct the formation of the appendages. Importantly, many of the signaling pathways and patterning genes identified and characterized in *Drosophila* have similar functions during vertebrate appendage development. In this review, we will summarize the genetic and molecular mechanisms that lead to the specification of appendage primordia in the embryo and their posterior patterning during imaginal disc development. The identification of the regulatory logic underlying appendage specification in *Drosophila* suggests that the evolutionary origin of the insect wing is, in part, related to the development of ventral appendages.

## 1. Introduction

Arthropods are the most successful group of animals and represent approximately three-quarters of the animal species currently living on Earth [1]. A key factor contributing to their success is their ability to exploit multiple ecological niches, most likely due to body plan modifications and innovations generating vast morphological diversity. Arthropod appendages show great variation in number, shape, and function, allowing multiple purposes from locomotion to feeding or environment sensing. However, besides their morphological disparity, it is likely that all appendages share common generative rules. Appendages have a proximodistal (P-D) axis that forms *de novo* orthogonal to the main body axes, the anteroposterior (A-P) and the dorsoventral (D-V). Molecular and genetic studies in the fruit fly, *Drosophila melanogaster,* have provided the basis of appendage development and served for comparative analysis with other arthropods [2]. Interestingly, many of the signaling pathways and patterning genes identified and characterized in *Drosophila* have comparable functions during vertebrate appendage development [3,4]. Thus, vertebrate and invertebrate appendages are built using similar underlying genetic programs, even though they are not homologous structures in the classic sense [5,6]. These similarities are often referred to as ‘deep homology’ [7].

Primitive insects develop appendages directly from embryonic limb buds that grow as external projections, while more derived insect species with complete metamorphosis, such as *Drosophila,* develop their appendages from imaginal precursors. The external adult body of *Drosophila* is formed by two different sets of cells, the imaginal discs and the histoblasts. The imaginal discs are specialized epithelial sheets specified in the embryo that grow and become patterned inside the larva. During the pupal stage, imaginal discs evert and differentiate to form the adult structures. The histoblasts are the precursors of the fly abdomen that, in contrast to the imaginal discs, only proliferate during pupal development. There are 19 imaginal discs in the larvae, 9 appearing in pairs, and the genitalia. The wing and haltere discs form the corresponding appendages and also the dorsal thorax. In a similar manner, the leg discs develop the appendage proper and the ventral pleura of the thorax. While thoracic imaginal precursors (wing, haltere, and legs) originate from a single embryonic segment [8,9], the genital disc primordia is a sexually dimorphic compound primordia derived from three abdominal segments (A8, A9, and A10) (reviewed in [10]). In a similar fashion to the genital primordia, the cells from different cephalic segmental identities coalesce together to form the eye-antennal disc [11]. The eye-antennal disc is also a compound structure that gives rise to the olfactory (antenna and maxillary palps) and visual (compound eyes and ocelli) organs plus the head epidermis [12]. We can group the appendages in dorsal or ventral depending on their relative positions within the body and their homology. Therefore, ventral appendages include the legs, antenna, and genitalia, while the wings and halteres are dorsal. In this review, we will focus on the patterning of the thoracic appendages and the antenna.

At the molecular level, a small number of signaling pathways are reiteratively used throughout development to specify and pattern the appendages in *Drosophila* and in vertebrates [3,4]. The function of signaling pathways such as hedgehog (Hh), decapentaplegic (Dpp), Wingless (Wg), Epidermal Growth Factor Receptor (EGFR) and Notch, and the transcription factors Distal-less (Dll), Homothorax (Hth) and members of the Sp family (Btd/Sp1) is fundamental in the formation of appendages. These signaling pathways and transcription factors are linked in regulatory networks, and cooperate to subdivide the developing appendages in different domains of gene expression. The identification and dissection of the *cis*-regulatory modules (CRMs) that controls the expression of genes required for wing, leg, or antenna development has been fundamental to decipher the regulatory networks that direct appendage formation [13,14,15,16,17,18]. Below, we discuss the regulatory logic behind appendage specification and patterning, and the implications in the evolutionary origin of the insect wing.

## 2. Allocation of the Thoracic Appendage Primordia

**Figure 1:**
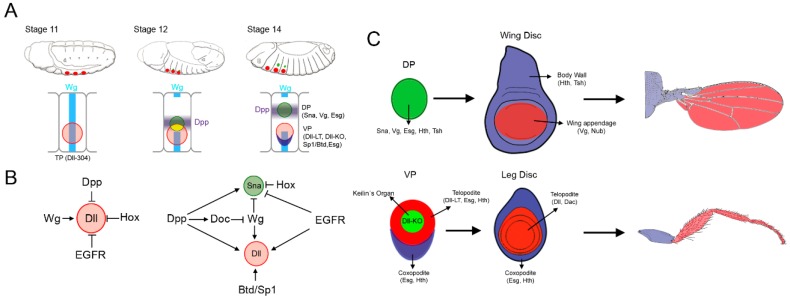
Specification of the thoracic appendages. (**A**) Cartoon showing three representative stages of *Drosophila* embryogenesis and the sequential appearance of the thoracic primordia (TP, red circle), the ventral primordia (VP, red circle) and the dorsal primordia (DP, green circle). Below is presented a schematic representation of the second thoracic segment where the expression of TP-, DP-, and VP-specific genes is related to the Wg and Dpp signaling pathways. Note that the DP originates from two populations of cells: one within the TP (drawn in yellow) and another one dorsal to the TP; (**B**) Genetic inputs that control appendage specification when *Dll* is first activated (stage 10–11) and during activation of the DP gene *sna* (stage 12 onwards). (**C**) Schematic representation of the DP and VP and the imaginal discs and adult structures that they will give rise to (wing and leg, respectively). The subdivision of the wing and leg imaginal discs in the body wall and appendage territories is also shown.

The development of imaginal discs is a progressive process initiated during embryogenesis with the specification of the imaginal primordia. Each primordium is formed by a characteristic number of cells located in the embryonic ectoderm in precise A-P and D-V location (Figure 1) [8,19]. All imaginal primordia are easily recognized by the restricted expression of the genes *escargot* (*esg*) and *headcase* (*hdc*), which maintain the imaginal state through the repression of endoreplication [20,21]. The segmental identity and position along the A-P axis of the different imaginal primordia is provided by the activity of Hox proteins [8,13,22,23,24]. The Hox genes encode homeodomain containing transcription factors that confer A-P identity along the body of the fly, defining unique developmental programs to each segment (reviewed in [25]). Therefore, the thoracic appendage primordia (legs, wing, and haltere) are restricted to their corresponding thoracic segments through the repression exerted by the abdominal Hox proteins (see below) [8,13,22,23].

The leg and wing/haltere presumptive imaginal discs are recognized in the thoracic segments of a late embryo as a genetically and morphologically distinct group of cells that form the ventral (VP) and dorsal primordia (DP), respectively (Figure 1A). The DP activates the expression of the *snail* (*sna*) and *vg* (*vestigial*) genes, while the VP expresses *Distal-less* (*Dll*) and the Sp family members *Sp1* and *buttonhead* (*btd*) [26,27,28,29,30]. The first molecular sign of appendage specification is the activation of the homeobox-containing gene *Dll* [5,8,19,31]. *Dll* is expressed in the outgrowths of many animals and is essential for appendage formation [5,32]. In *Drosophila*, *Dll* expression is initiated in a group of about 30 cells in each thoracic hemisegment at stage 10 of embryogenesis [19]. Multiple *Dll* CRMs have been identified that partially reproduce the spatial and temporal expression of *Dll*. The characterization of these *Dll* CRMs reveals not only the molecular logic of *Dll* regulation but also allows the description of the developmental fate of *Dll* expressing cells [14,15,16,23]. At stage 10–11, an early *Dll* CRM, named *Dll*-304, is activated in a pattern similar to *Dll* and its activity decays some hours later [8,23]. These cells are defined as thoracic primordia (TP) because their progeny will contribute to both the VP (leg) and DP (wing/haltere) (see below). At this stage, *Dll* expression is positively regulated by Wg, that is expressed in D-V stripes in the anterior compartment of each thoracic segment [19]. The precise localization of *Dll* expression along the D-V axis in the thoracic embryo epidermis is also regulated by repression mediated by Decapentaplegic (Dpp) and the Epidermal Growth Factor Receptor (EGFR) pathways in dorsal and ventral cells, respectively [33] (Figure 1B). The regulation exerted by the Wg, Dpp, and EGFR signaling pathways is present in the thoracic and abdominal segments, although *Dll* expression is restricted to the thorax by repression mediated by the abdominal Hox proteins [23,34,35]. The Hox proteins Ultrabithorax (Ubx), Abdominal-A (Abd-A), and Abdominal B (Abd-B) directly bind to the early *Dll*-304 CRM in a compartment specific manner [35]. Although *Dll* could be activated in the absence of Antenapedia (Antp), the thoracic Hox protein, it has been recently shown that Antp plays a positive role in enhancing *Dll* expression levels [36].

### 2.1. Specification of the Ventral and Dorsal Primordia

Classic lineage experiments using X-ray somatic recombination and gynandromorphs already suggested that the dorsal and ventral primordia originated at the blastoderm stage in close proximity and presumably derive from an overlapping population of cells [37,38,39]. In accordance, more recent lineage-tracing experiments revealed that the progeny of *Dll* or *Dll*-304 expressing cells (TP) not only contribute to the entire leg imaginal disc, including the ventral body wall and the appendage, but also to the wing disc [13,16]. The initial group of *Dll* expressing cells (TP) is subdivided as embryogenesis progresses in cells that will contribute to the DP (wing and haltere) and the VP (legs). This process requires a correct balance between the D-V positional cues provided by the Dpp, EGFR, and Wg pathways [8,13,33,40,41] (Figure 1A,B). *dpp* expression at the dorsal side of the TP field generates a concentration gradient that promotes DP fate at high levels and specifies the VP fate at intermediate levels [33]. Simultaneously, the ventral activation of the EGFR pathway antagonizes Dpp signaling and restricts the activation of the wing promoting genes to the dorsal domain of the TP [40]. Dpp also activates the *Dorsocross* (*Doc*) genes in a broad lateral stripe that partially overlaps with the dorsal domain of the TP [42]. This leads to the repression of *wg* expression in the lateral ectoderm [13,43,44] (Figure 1A,B). In this manner, dorsal TP cells are exposed to high levels of both Doc and Dpp signaling and low Wg and EGFR. These cells will contribute to form the DP, lose *Dll* expression and activate the DP developmental program. In contrast, ventral TP cells retain high levels of Wg and EGFR, maintain *Dll* expression, and are fated to form the VP. Contrary to the opposite roles that the Dpp and EGFR pathways have on DP formation, both pathways positively direct VP fates [8,13,40,41]. At this stage, the role of the Wg pathway is restricted to VP, as it is necessary for *Dll* expression but not for wing formation [41].

The repression of Wg and the presence of high Dpp levels are a prerequisite for the activation of the DP promoting genes *sna*, *esg*, and *vg* in cells of the lateral ectoderm and dorsal to *Dll* expressing cells (Figure 1A) [13,33,40,41]. The activation of *sna* and *vg* is only observed in Dll-negative cells after the separation of the dorsal and ventral primordia. In contrast to *Dll*, which is expressed in the three thoracic segments, the activation of the DP specific genes (*sna*, *esg*, and *vg*) only occurs in the second and third thoracic segments (T2 and T3). *sna* and *esg* act redundantly to specify DP fate while *vg* is required later for the development of the wing and haltere appendages but not for DP formation [27,28]. At the end of embryogenesis, the different size of the wing and haltere primordia is already apparent, being the haltere approx. 30% smaller than the wing primordia. The number and size of the DP is regulated by the Hox proteins [13,22]. Restriction of the DP to the T2 and T3 segments is controlled by the repressor activity of Sex combs reduced (Scr) in T1 and by Ubx, Abd-A, and Abd-B in the abdominal segments [13,22]. Remarkably, Ubx has two different functions in DP specification: in the abdominal segments represses DP formation, while in the third thoracic segment regulates the size of the haltere. How Ubx exerts these two functions is unknown, but could be possibly explained by the different Ubx levels observed between the T3 and A1 segments [13,22,24]. High levels of Ubx in the A1 could repress DP genes while lower levels in T3 would reduce, but not eliminate, their activation. Another possibility is that the different behavior of Ubx would be explained by the use of diverse Hox cofactors in each of these segments.

### 2.2. Dual Developmental Origin of the Drosophila Wing and Its Evolutionary Implications

The origin of the insect wing is one of the most intriguing evolutionary events in biology, and is still under debate. Two main theories have been proposed to explain the origin of this morphological innovation (reviewed in [45]). The paranotal or tergal hypothesis proposes that wings are created *de novo* as an extension of the dorsal thorax or tergum [46]. The alternative theory, the gill-exite or pleural hypothesis evolutionarily connects the wings to a preexisting structure, the gill, present in the proximal base of an ancestral leg [47,48,49]. Studies in *Drosophila* and other insects have contributed significantly to decipher the origin and specification of insect appendages [8,44,50,51,52,53]. Recently, thanks to the identification and characterization of the *sna* dorsal primordia CRM (*sna*-DP), a clear picture of the origin and the genetic inputs that control DP specification was obtained [13]. *sna*-DP is first activated in a few cells dorsal to *Dll* expressing cells in T2 and T3 that later label the entire DP. A cell lineage analysis of *sna*-DP cells demonstrated that this CRM labels the entire wing disc cells including the progenitors of the thorax and appendage. Importantly, cells that have expressed—but no longer actively transcribe—*Dll* populate half of the DP [8,13,41]. These cells can be located at any part of the wing disc, although they are found preferentially in the ventral domain of the disc [13,16]. This bias is probably due to the relative location of the DP in relation to the VP in the embryo rather than to a preexisting genetic determination of these cells. In addition, genetic ablation of the TP domain reduces, but does not eliminate the *sna*-DP cells, suggesting the existence of DP cells that form independently of the TP [13]. Moreover, DP fates could be specified in the absence of *Dll* or the ventral appendage selector genes *buttonhead* (*btd*) and *Sp1* [8,13,41,54]. All these experiments and the lineage tracing analysis indicate that the DP is derived from two populations of cells: one that derives from early *Dll* expressing cells (TP), and another that arises close to, but is independent of *Dll* expressing cells (Figure 1A). Importantly, as discussed above, both populations of cells that conform the DP require the same molecular signals for their specification: high levels of Dpp and low Wg [13] (Figure 1B). This dual developmental origin of the wing described for *Drosophila* is in accordance with recent evo-devo studies that support the unification of the paranotal and pleural hypotheses [44,50,51,53,55].

### 2.3. Proximo-Distal Subdivision of the Ventral Primordia

In contrast to the DP, where distinction between trunk and appendage occurs later in imaginal development (see below), the VP is organized in gene expression domains that subdivide the primordia in different territories (Figure 1C) [16,56,57,58]. The development of the VP requires the function of *Dll* and of the two paralogous genes *btd* and *Sp1*. Btd and Sp1 are members of the highly conserved Sp family of transcription factors required for appendage formation in vertebrates and invertebrates [30,59,60,61,62,63]. *btd/Sp1* are expressed in the embryonic progenitors of the leg imaginal discs starting around stage 10-11 [29,30,54,64]. As it is the case with *Dll*, *btd* is activated by Wg and repressed dorsally and in the abdominal segments by Dpp and Ubx, respectively [30]. At stage 11, *btd*, *Sp1* and *Dll* (*Dll*-304) are activated in parallel in the TP and are genetically independent of each other. Importantly, as mentioned before, these TP cells could contribute to the DP and to the entire VP. Some hours later, at stage 14, VP and DP are already separated and the activity of the *Dll*-304 CRM decays. At this time point, *Dll* expression is controlled by two *Dll* CRMs with mutually exclusive patterns at the VP, the leg trigger (*Dll*-LT) and the Keilin organ (*Dll*-KO) elements (Figure 1C) [16]. The progressive refinement of the appendage primordium developmental potential is reflected by the *cis*-architecture of the *Dll* gene [14,16,65]. At the end of embryogenesis, the VP is genetically subdivided in domains with different cell fates that correspond to the P-D subdivision of the arthropod leg proposed by Robert E. Snodgrass (see below) [66,67] (Figure 1C). VP cells that express *esg* but do not activate *Dll* will form the coxopodite, the most proximal and unsegmented domain of the appendage that forms as an outgrowth of the bodywall, including the coxa. VP cells co-expressing *esg* and *Dll* (through the *Dll*-LT CRM) will form the telopodite or the leg proper, which includes all the distal leg segments that are articulated. Additionally, cells at the center of the VP that are *esg* negative and Dll positive (*Dll*-KO) are fated to form a larval mechanosensory structure that shares a common lineage with the leg disc called the Keilin’s organ (KO) [16,67]. While *Dll*-KO cells do not contribute to the leg imaginal disc, the progeny of *Dll*-LT activating cells form the entire telopodite. Consistently, in *Dll* mutants the telopodite is lost while the coxopodite is present [8,16,68,69]. *Dll*-LT is positively regulated by Wg and Dpp and repressed in the center of the VP by members of the *achaete–scute complex* (ASC) [14,16,67]. At the same time, Dll and ASC positively regulate the *Dll*-KO CRM restricting its activity to the VP [16]. At this stage *btd* and *Sp1* act upstream of *Dll* and only the elimination of both genes suppresses *Dll* expression and the activity of the *Dll*-LT and *Dll*-KO enhancers [14,16,54]. Initially, some studies proposed that the two related homeobox genes Homothorax (Hth) and nuclear Extradenticle (Exd) were markers, along with *esg,* of coxopodite fate [33,40,41,70,71]. However, a reevaluation of the expression of *hth*, *esg* and *Dll* and their specific cell fate helped define a high-resolution map of the VP fates formed by three molecularly different domains with distinct developmental potential [16,67].

## 3. Specification of the Appendage Domain and Patterning of the Leg, Antenna and Wing Imaginal Discs

After the specification stage in the embryonic ectoderm, each imaginal primordium follows a developmental program in which the same signaling pathways and an increasing number of transcription factors cooperate to pattern the growing epithelium. In this process the size of each disc increases by cell proliferation, and the epithelium is progressively divided into domains of gene expression that confer each territory with particular cell identities. A common aspect of all thoracic discs is their division in A and P compartments conferred by the expression of *engrailed* (*en*) in posterior cells. Compartment boundaries are the source of signaling molecules that organize the pattern and growth of the imaginal disc [72]. In this section we will describe the molecular mechanisms that distinguish between trunk and appendage and the subsequent patterning of appendages culminating in the assignation of cellular fates that will specify the future domains of the adult organs.

### 3.1. Patterning of the Leg Imaginal Disc

**Figure 2:**
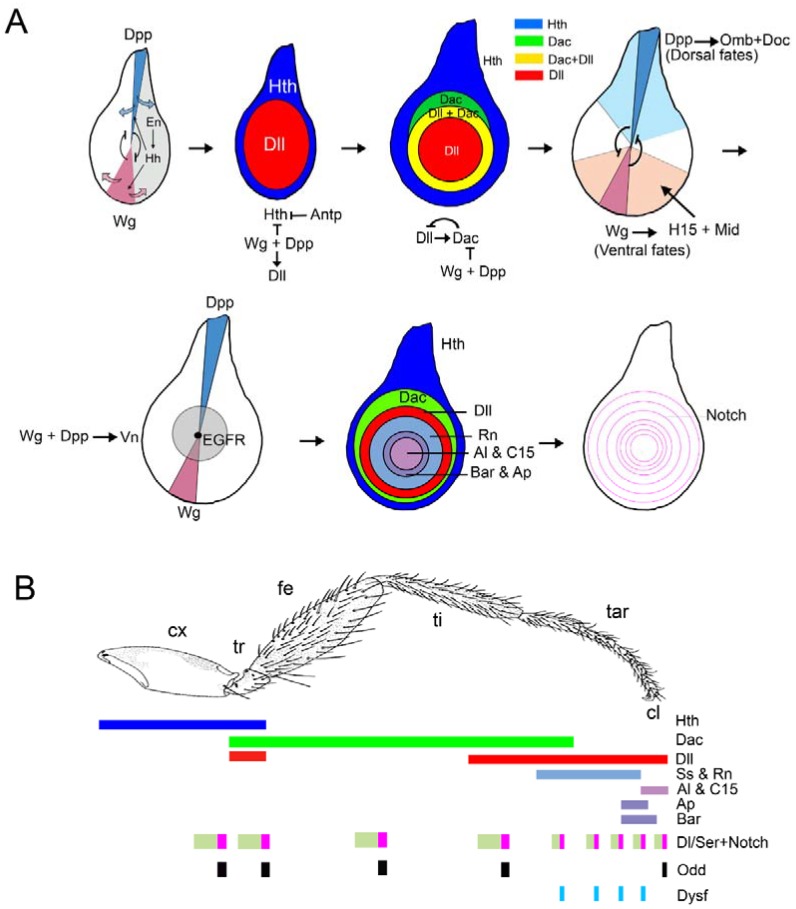
Patterning of the *Drosophila* leg. (**A**) Overview of the proximo-distal (P-D) and dorso-ventral (D-V) axes formation during leg development. The restricted expression of *dpp* and *wg* in the dorsal and ventral halves of the leg disc is required to initiate and to pattern the P-D and D-V axes. The first two leg imaginal discs from the left correspond to early second instar discs, while the rest depict third instar leg discs. Some of the genetic interactions that lead to the leg patterning are indicated. (**B**) The ‘genetic code’ generated by the expression of several transcription factors specify the future segments of the adult leg along the P-D axis. The expression of the Notch ligands *Dl* and *Ser*, and the activation of the Notch pathway and its target genes is necessary for the formation of the joints that separate the adjacent segments of the leg.

The third instar leg imaginal disc is roughly circular in shape with the distal-most region of the leg located in the center of the disc, and the most proximal leg segments and the pleura arising from the periphery of the disc. *Antp* is the selector Hox gene for leg identity that is expressed in the leg imaginal disc but not in the antenna disc. As expected for its selector function, *Antp* mutant legs are transformed into antenna and *Antp* ectopic expression in the antenna converts it into leg [73]. The leg P-D axis is formed orthogonally to the previously established A-P and D-V axis, and its formation is intimately connected with the A-P compartment subdivision. Thus, posterior cells express *hedgehog* (*hh*) which acts as a short-range signal that activates the expression of *dpp* and *wg* in the anterior dorsal and ventral halves of the disc, respectively [74]. Dpp and Wg are both necessary to establish and pattern the D-V and the P-D axes. The D-V axis is organized by the mutually antagonistic repression of Wg and Dpp and interactions between its downstream genes (Figure 2A) [75,76,77,78,79,80]. Briefly, Wg specifies ventral fates through the activation of *H15* and *midline* (*mid*) and the repression of dorsal genes such as *Doc* and *optomotor blind* (*omb*) [43,81]. Therefore, in *wg* mutants all the ventral structures are lost and replaced by a mirror duplication of dorsal ones and the reverse phenotypes are observed for *dpp* mutants [75,76,78,80,82].

The P-D axis is initiated at the center of the disc where high levels of Wg and Dpp activate downstream genes such as *Dll* [14,83,84,85,86]. Dll in turn activates *dachshund* (*dac*) in the medial domain of the leg disc, whereas high levels of Wg and Dpp represses its expression in the distal tip [85,87]. Once activated, *Dll* and *dac* expression is maintained in part by autoregulatory mechanisms [14,87]. Simultaneously, the expression of *hth* is restricted to the periphery of the disc by a Wg and Dpp dependent repression mechanism [88,89]. This mechanism could be mediated by the zinc finger proteins encoded by the *elbow* (*el*) and *no ocelli* (*noc*) genes that act in the leg and the wing discs to repress *hth* expression, and therefore body wall fates [90]. Consequently, as a result of the A-P compartment subdivision and the restricted expression of Hh, Dpp, and Wg the leg is divided along the P-D axes in proximal, medial, and distal domains defined by the expression of *hth*, *dac*, and *Dll,* respectively (Figure 2A). At this stage *Antp* prevents *hth* and *Dll* coexpression in the leg, suppressing antennal identity [91,92,93,94]. Importantly, the ancestral subdivision of the leg in the coxopodite and telopodite is reflected at the molecular level by the differential response of the leg imaginal disc cells to the Wg and Dpp signaling pathways. Therefore, cells that receive both Wg and Dpp activate the *Dll* and *dac* genes and specify the telopodite. In contrast, cells that are unable to respond to these pathways are specified as coxopodite and activate *hth* [70,71] (Figure 1C).

The distal domain of the leg is further patterned by a P-D gradient of EGFR signaling, mediated by the EGFR ligand Vein (Vn), that regulates the expression in a distal to proximal manner of *aristaless* (*al*), *Bar* (*B*), and *rotund* (*rn*) among others [95,96] (Figure 2A,B). A complex cross-regulation between these transcription factors ensure the segmental subdivision of the distal domain in five tarsi (reviewed in [97,98]). Besides providing unique segmental identity along the P-D axis of the leg, this regulatory network is responsible for the segmentally repeated expression in concentric rings of the Notch ligands *Delta* (*Dl*) and *Serrate* (*Ser*) [99,100]. Notch activation at the distal-end of each presumptive segment is evident at the end of larval development and directs the formation of the joints and the non-autonomous growth of the leg [101,102,103] (Figure 2A,B). The ventral appendage selector-like genes *btd* and *Sp1* are co-opted later during imaginal development to control the growth and pattern of the leg, in part through the regulation of *Ser* expression and therefore Notch signaling [100]. Two types of joints with different morphologies and evolutionary origin could be found in the adult leg. The proximal or true joints are asymmetrical and have associated musculature, whereas the distal joints are symmetrical and not attached to muscles [66,104,105]. Notch controls joint morphogenesis through the activation of subsidiary transcription factors required for distal joints such as *dysfusion* (*dysf*), proximal joints such as the *odd-skipped* gen family of for all joints like *dAp-2* ([103,106,107] and reviewed in [108]).

### 3.2. Patterning of the Wing Imaginal Disc

**Figure 3:**
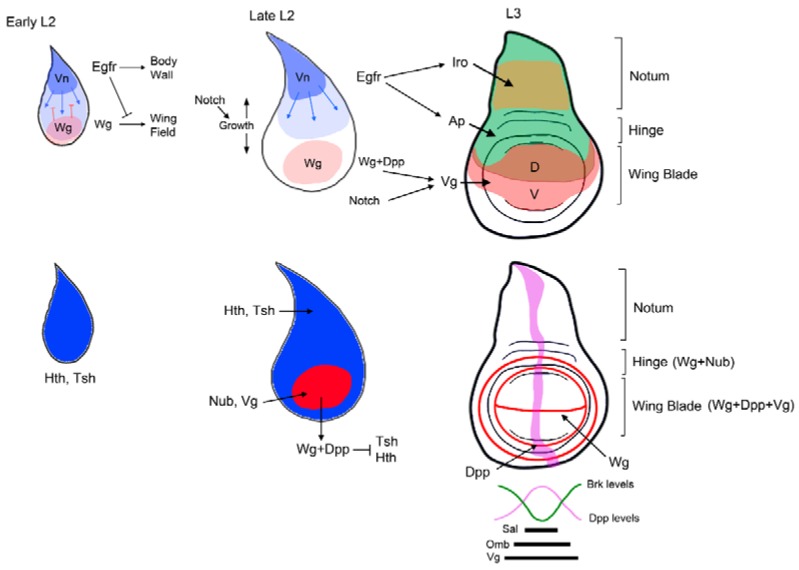
Patterning of the *Drosophila* wing. Three representative stages of wing disc development are depicted. The signals and genes that direct the specification and patterning of the appendage (wing) versus the body wall are illustrated above. The activity of the EGFR pathway promotes body wall identity while repressing wing fates. Notch induces the growth of the wing disc, which separates the source of the Wg and EGFR pathways and allows the specification of the wing field by Wg and the activation of wing promoting genes. Below are shown the expressions of the Dpp and Wg pathways in a third instar disc and the relative position of the Dpp target genes *sal*, *omb*, and *brk*.

The DP contains the progenitors of both the wing and the body wall structures, however in contrast to early P-D specification of the VP, the trunk/appendage distinction is only evident later during larval development (Figure 1C and Figure 3). The mature wing disc is formed by two distinct domains, the body wall and the appendage, which in turn is subdivided in the proximal hinge and the wing blade. The specification of the body wall and the wing appendage is made by the mutually repressive activities of two signaling pathways: the Vn/EGFR acting in the proximal domain of the disc that specifies body wall fates, and Wg at the distal region that will form the wing field (Figure 3) [109,110,111,112].

Consequently, in the absence of Wg the wing field is lost and a duplication of proximal structures is observed [56,58]. In the early primordium, the activity of the EGFR pathway represses the ability to induce wing fates by Wg and promotes notum fates [110,111]. As the disc grows in size induced by the activity of Notch, the signaling sources of the EGFR and Wg pathways become separated allowing the specification of the wing field by Wg and the activation of wing promoting genes such as *nubbin* (*nub*) and *vg* [58,113]. The activation of *nub* and *vg* is accompanied by the repression of the body wall genes *teashirt* (*tsh)* and *hth* (Figure 3) [114,115]. The different P-D expression of *hth/tsh* (notum and hinge) and *vg* (wing blade) is analogous to the leg disc, where *hth/tsh* are also restricted to the proximal domain to promote body wall structures (Figure 1C). It has been proposed that as in the leg, Wg and Dpp in the wing disc are required, through the activation of *el* and *noc,* to repress *hth* and *tsh* expression from the wing territory [90,114,116]. However other mechanisms have also been proposed [115]. The EGFR pathway is also required to activate the dorsal selector gene *apterous* (*ap*) that subdivide the thorax and the wing field in dorsal (D) and ventral (V) compartments [17,111,117,118]. This D-V subdivision is absolutely necessary for *vg* expression and wing outgrowth [119]. Ap regulates the expression of the Notch ligands Dl and Ser that in turn activate the Notch pathway at the D-V boundary. Wg and Notch cooperate to induce the expression of *vg* in the cells that will become the wing blade [120,121,122]. The mutual antagonism between *hth* and *vg* subdivide the wing field in the pouch and the hinge [116]. The hinge region of the wing field is specified by the combined action of Wg, Hth, and Tsh [92,114,121]. In the proximal region of the disc, the EGFR pathway induces the expression of the notum specifying genes of the *Iroquois-complex* (*Iro-C*) that are repressed in the wing field by Dpp secreted in the A-P boundary (Figure 3) [123,124,125,126]. Once specified, the notum is further subdivided in medial and lateral domains by the activity of the transcription factor Pannier (Pnr) that promotes medial *vs* lateral fates [127].

As in the leg disc, *en* expression subdivides the wing disc in two populations of cells, the A and P compartments. The A-P boundary acts a source of positional information where Hh secreted from the posterior compartment activates the expression of *dpp* in A cells. Dpp and Hh are both required to pattern the wing blade along the A-P axis (Figure 3). Dpp diffusion from the A-P boundary restricts the expression of *brinker* (*brk*) to the lateral domain of the wing [128,129,130]. Dpp and Brk in turn, regulates the expression of *omb* and the *spalt* genes (*sal*) in nested domains [130]. The Hh and Dpp signals act coordinately with the EGFR signaling pathway to position and maintain vein territories in the developing wing (reviewed by [131,132]).

### 3.3. Patterning of the Eye-Antennal Disc

**Figure 4:**
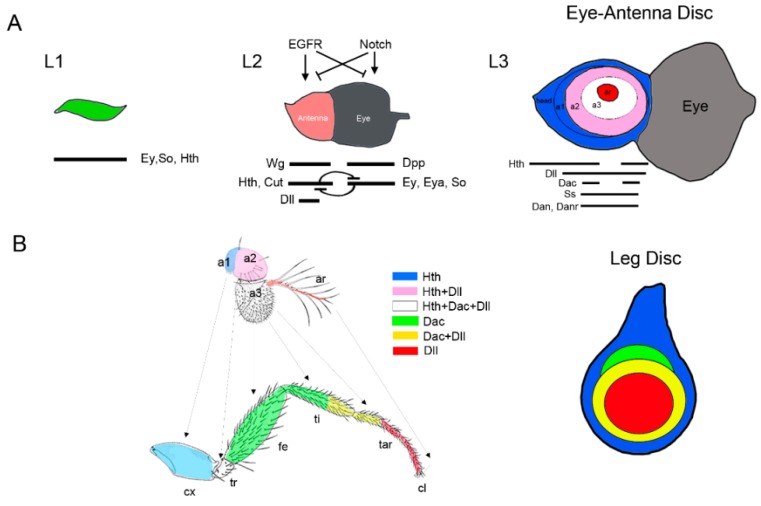
Specification and patterning of the *Drosophila* antenna. (**A**) Model of progressive specification of the eye and antenna territories and P-D subdivision of the antenna disc. Schematic representation of three representative stages of EAD development is shown. In each disc the expression and interactions of genes and signaling pathways required for eye and antenna field specification are schematized. (**B**) The antenna and leg appendages are homologous structures. Arrows indicate the correspondence between the antenna and leg domains (Postlethwait and Schneiderman, 1971). The expression of the P-D genes *Dll*, *dac*, and *hth* and their overlapping domains are represented by a color code. Compare the relative expression of *Dll*, *dac*, and *hth* in the antenna and the leg imaginal discs. Note that *hth* and *Dll* coexpress in a large domain in the antenna while in the leg these genes are expressed in almost exclusive domains.

The eye-antennal disc (EAD) is formed by different populations of cells located in the cephalic segments that coalesce together [11]. The EAD is a compound disc that gives rise to the eye, the ocelli, the palpus and the antenna plus the head cuticle that surrounds them. Homeotic transformations from antenna to leg have been described for several mutations, including *hth*, *spineless* (*ss*), and *Antp* that converts every antenna part to its corresponding leg segment [133,134] (Figure 4B). Therefore, the antenna and leg appendages have been considered serial homologous structures that share a similar developmental program. Thus, variations in this developmental program are responsible for the morphological differences between these two appendages.

Early on larval development (L1) the EAD shows no sign of regional specification and presents uniform expression of several transcription factors including *hth* and *tsh* and the eye determinants *eyeless* (*ey*) and *sine oculis* (*so*) (Figure 4A) [135,136,137]. The two main developmental fields within the disc, the antenna and the eye, are segregated during larval stages (L2) at the time when specific eye and antenna determinants are restricted to the corresponding EAD domain [136,137,138,139,140]. The transcription factors Ey, Eyes absent (Eya) and So are selectors for eye development while Hth, Dll, Ss, and Cut have been proposed to select for antennal fates. The antagonistic interactions between the Notch and the EGFR pathways regulate the eye and antennal fate decision [135] (Figure 4A). Accordingly, the downregulation of the Notch pathway or the activation of the EGFR in the eye field transforms it into an antenna [135]. However, the role of Notch as an eye identity inducer has been debated [136]. In this view, Notch influences eye field specification through its control on cell proliferation, thus modulating the activity of the Dpp and Wg signaling pathways located at opposing sides of the early EA disc [136,141]. Wg is a negative regulator of eye development while Dpp induces the expression of the eye determinant *eya* ([136,142] and reviewed in [143]). Once the two morphogenetic fields have been established, the restricted expression of antenna and eye promoting genes is observed (Figure 4A). For example, Cut and Hth represses the eye specification genes *ey* and *so* while the latter represses *hth* and *cut* [137]. The patterning logic that controls the P-D axis formation in the antenna is similar to what can be found in the leg [84]. Hh secretion from posterior cells activates the expression of *dpp* and *wg* in dorsal and ventral anterior cells, respectively that in turn directs P-D axis formation through the regulation of *Dll*, *Dac*, and *hth*. As in the leg, Dll specifies distal fates while Hth promotes proximal identities. However, in contrast to the leg where the domains of Dll+Dac and Hth are mostly exclusive, these genes are co-expressed in a large territory in the antenna (Figure 4A) [91,144,145]. The presence in the leg of the selector gene Antp is a key factor for *hth* repression while its absence in the antenna allows *Dll* and *hth* coexpression [91,94,146]. Antennal identity depends on the coordinated action of Hth and Dll, which together activate the expression of antennal determinants such as *ss or spalt* (*sal*) [145,147]. Ss in turns control the expression of *distal antenna* (*dan*) and *distal antenna related* (*danr*) genes, responsible for the specification of distal antenna identity [148,149].

## 4. Perspectives

In this review, we have discussed how the study of *Drosophila melanogaster* has provided a detailed picture of the molecular mechanisms that lead to the specification and patterning of the adult appendages. Importantly, many of the signaling pathways and transcription factors identified in the fly have similar functions during vertebrate limb development. However, many important questions remain unsolved. First, the target genes and the regulatory networks controlled by the transcription factors that subdivide the appendages along the different axes are still largely unknown. This includes the analysis of the molecular interactions between patterning genes and signaling pathways. Second, further investigation would be required to unravel how the patterning information is translated into cellular behaviors (cell growth, division, and cytoskeleton organization, among others), which are ultimately responsible for the characteristic shape and size of the different appendages of the fly. The use of whole genome techniques such as ChIP (Chromatin Immunoprecipitation) or ATAC (assay for transposase accessible chromatin) assays, coupled with whole genome sequencing is essential to identify the target genes and the regulatory landscapes governed by the patterning transcription factors. In addition, live imaging techniques would provide novel information of the morphogenetic processes and dynamics during appendage specification and formation.
